# Incidents and Potential Adverse Health Effects of Serious Food Fraud Cases Originated in Asia

**DOI:** 10.3390/foods12193522

**Published:** 2023-09-22

**Authors:** Varongsiri Kemsawasd, Vijay Jayasena, Weeraya Karnpanit

**Affiliations:** 1 Institute of Nutrition, Mahidol University, 999, Salaya, Phutthamonthon, Nakhon Pathom 73170, Thailand; 2 School of Science, Western Sydney University, Locked Bag 1797, Penrith, NSW 2751, Australia; v.jayasena@westernsydney.edu.au

**Keywords:** adulteration, food fraud, food hazard, fraudulent, health risk

## Abstract

Food fraud has long been regarded as a major issue within the food industry and is associated with serious economic and public health concerns. Economically motivated adulteration, the most common form of food fraud, has consequences for human health, ranging from mild to life-threatening conditions. Despite the potential harm and public health threats posed by food fraud, limited information on incidents causing illness has been reported. Enhancing the food control system on the Asian continent has become crucial for global health and trade considerations. Food fraud databases serve as valuable tools, assisting both the food industry and regulatory bodies in mitigating the vulnerabilities associated with fraudulent practices. However, the availability of accessible food fraud databases for Asian countries has been restricted. This review highlights detrimental food fraud cases originating in Asian countries, including sibutramine in dietary supplements, plasticizer contamination, gutter oil, and the adulteration of milk. This comprehensive analysis encompasses various facets, such as incident occurrences, adverse health effects, regulatory frameworks, and mitigation strategies.

## 1. Introduction

Asian countries, especially China and India, are significant contributors to the global food system because of their large populations [1,2]. The Asian continent is home to more than half of the total global population; therefore, strengthening the food control system of this continent is important for international health and trade. Food fraud can occur at any point of the food supply chain, from farm to table [3]. Food fraudulent practices have a considerable impact on the domestic economy and international trade. In the modern trade era, incidents of food fraud in one country can easily become an international emergency affecting global health and trade because of rapid and widespread food distribution networks [4]. Food fraud activities include, but are not limited to, concealment, counterfeiting, misrepresentation, substitution, and unapproved enhancement [4]. False or misleading statements about a food product are also considered fraudulent [3,5,6].

Databases gathering information on historical and current food fraud incidents have been developed for decades. Food fraud databases can be used as tools to assist the food industry and regulators to reduce the risks of food fraud. There have been several food fraud databases developed, including the United States Pharmacopeial Convention (USP) Food Fraud Database, the National Center for Food Protection and Defense (NCFPD) Economically Motivated Adulteration (EMA) Incident Database, the European Commission’s Rapid Alert System for Food and Feed (RASFF), and the Food Adulteration Database (FADB)—China. However, only a few databases, such as the RASFF and the FADB, are open and publicly accessible. The USP Food Fraud Database currently belongs to FoodChain ID Group Incorporated and the NCFPD EMA Incident Database is currently owned by FoodSHIELD; these databases are not freely available to the public. Although these databases may not be comprehensive, they provide useful information to gain further insight into the scope of food fraud, as a means for detecting or preventing future frauds [7].

The RASFF was established to mitigate food safety issues through a centralized reporting procedure. As a response to food fraud occurrences, the RASFF identified “fraud and adulteration” as one of the food hazard categories [8]. The RASFF method has been widely accepted as a trusted source of information, involving information exchange facilitated by experts. In this review, alert notifications of food fraud and adulteration cases specific to Asia were analyzed over a ten-year period from 2011 to 2020. The RASFF portal database reported 1166 cases under the “fraud and adulteration” hazard category, where 663 cases (56.9%) were from food products of Asia. Over 70% of the Asian-originated food frauds and adulterations were discovered in China (200), India (172), and Turkey (117), as shown in Table 1. Nuts, nut products, and seeds were the most frequently reported food products with adulterations (189 cases), followed by fruits and vegetables (96 cases), and herbs and spices (89 cases). Health certificates were documented in only 440 cases. There was an absence of health certificates for 279 reported cases, while 99 cases included an improper health certificate, 52 cases found fraudulent health certificates, and one case involved an invalid health certificate (Table 2). Owolabi and Olayinka (2021) investigated the incidents of food fraud and adulterations (FFA) in foods imported into the European Union from the Association of Southeast Asian Nations (ASEAN) [9]. The food fraud occurrences were extracted and analyzed from the RASFF and Food Fraud and Adulterations (FFA) databases from 2000 to 2020. Among the 10 ASEAN member countries, the highest number of food fraud cases were found in foods imported from Thailand (47 cases), followed by the Philippines (37 cases). Herbs and spices were the products imported from this region with the highest incidents of food fraud and adulteration [9].

Recently, a molecular-level food adulteration database has been established for predicting the presence of illegal food additives based on molecular fingerprints and structural similarities. A compilation of 961 cases between 1998 and 2019 from the published information were included in the database. Food fraud prediction based on the database resulted in the identification of 1919 illegal chemicals that may be added to foods. More than 130 kinds of adulterated foods have been included in the FADB. The database is an effective tool for the detection and prevention of emerging food frauds [10].

Food fraud incidents in Asia and fraudulent products originated in Asian countries which have imposed serious health threats, namely sibutramine in dietary supplements, the plasticizer scandal, gutter oil, and adulterated milk, were reviewed with regard to incidents, public health impact, regulations, and mitigation approaches.

## 2. Addition of Sibutramine to Dietary Supplements for Weight Loss

One of the most serious issues found in food fraud cases was the adulteration of food supplements with sibutramine, an active synthetic substance, to enhance weight loss [11,12]. Sibutramine is a prescription medication for weight loss for overweight and obese patients, with a recommended daily dose of 5 to 15 mg [13]. Sibutramine is a serotonin–noradrenaline reuptake inhibitor (SNRI) structurally similar to amphetamine [13,14]. Side effects of sibutramine include xerostomia, insomnia, headache, numbness, paresthesia, nausea, anxiety, and constipation. This substance is also linked to detrimental effects on the cardiovascular system, including an elevated blood pressure and pulse rate, with increased risks for acute heart disease and cardiac arrest [12,13,14].

The quantity of sibutramine found a Chinese herbal weight-loss medicine sold over the internet was approximately two times higher than the maximum recommended dosage [12,14]. There have been reports demonstrating an association between sibutramine and psychosis, mania, and panic attacks. This may have been due to the mechanism of dopamine reuptake inhibition, leading to psychotomimetic effects with regular intake [15,16,17]. A study found 17 case reports of health problems in Germany from the intake of Chinese slimming capsules [13]. Symptoms ranged from malaise and arterial hypertension to acute psychosis. The weight loss supplements collected from 2005 to 2008 contained high levels of sibutramine: 32.7 and 28.3 mg, respectively. Sibutramine-adulterated slimming products of Chinese origin which were sold over the internet have caused a range of issues, from severe fatal outcomes to mild intoxications [11]. Cardiac arrest was reported in a young Japanese woman taking a weight loss supplement containing sibutramine imported from Thailand. The patient had no history of cardiovascular disease, and she had taken the supplement only once [18]. One case study reported an association between sibutramine intake and sudden cardiac death in a patient with no previous coronary heart disease history. The patient had taken weight loss herbal supplements, and sibutramine and its metabolites (desmethylsibutramine and didesmethylsibutramine) were found in the serum. “Complications of Acute Sibutramine Intoxication” were identified as the cause of death [19]. Table 3 shows the levels of detected sibutramine in dietary supplements. Sibutramine concentrations in various weight-loss supplements collected in Asian countries varied considerably, from 0.14 to 781,200 mg/kg. High levels of sibutramine (5400–781,200 mg/kg) were found in nine slimming capsules purchased from unauthorized stores in Iran. These food supplements were manufactured in Asian countries, including China, Iran, and Southeast Asian countries.

Reports of serious health effects associated with herbal supplements have contributed to an understanding of the significance of developing an effective system for the inspection and declaration of product composition. China adopted the Chinese Food Safety Law in 2015, with a new system to regulate dietary supplements. Products “Classified as Supplements” must undergo extensive testing, pre-market approval, and toxicity testing prior to sale [20]. Japan’s 2015 legislation divides “Food with Health Claims” into three categories: “Food with Nutrient Function Claims” (FNFC), “Food Specified for Health Uses” (FOSHU), and “Food with Function Claims” (FFC). Health foods may only be marketed as FOSHU after approval from the Consumer Affairs Agency. The process requires the submission of label claims, along with the provision of evidence on the safety and efficacy of the product [20]. For FFC, manufacturers must submit information on safety, effectiveness, and the system for information collection on adverse health effects to the Consumer Affairs Agency. Unlike FOSHU, the safety and effectiveness of FFCs are not evaluated by the government [21].

While a standardized approach for herbal products is being developed across the world, at present, there is no common practice or understanding [22]. Regulations on natural supplements are inconsistent across countries, creating the need for collaboration among regulatory agencies to improve the global standards for natural supplements [23]. Enforcing the obligation to declare ingredients and the appropriate dose is a crucial step towards transparency and consumer safety [13]. Strict controls on slimming product regulations, including licensing, labeling guidelines, and ingredient verification, may be required to combat fraudulent acts [12].

**Table 3:** Detection of sibutramine-adulterated food supplements from different Asian countries.

Country of Sample Collection	Sample Collection Duration	Detection Rate as Percentage (Positive Sample/Total Sample)	Sibutramine Concentration ^a^ Min–Max (mg/kg)	Reference
United Arab Emirates	NA	15.3% (21/137)	0.14–16,823	Jairoun et al., 2021 [24]
South Korea	2009–2012	25.5% (48/188)	30–132,400	Kim et al., 2014 [25]
Singapore	2012–2014	12.3% (55/447)	NA	Zeng et al., 2016 [26]
Vietnam	NA	31.6% (6/19)	1.11–14,850	Hieu et al., 2021 [27]
Iran	2019–2020	27.0% (17/63)	4.38–26.37 mg/capsule	Firozian et al., 2021 [28]
Turkey	NA	33.3% (3/9)	35,000–45,000	Ozdemir et al., 2013 [29]
South Korea	2015–2017	2.7% (10/370)	9900–135,000	Yun et al., 2017 [30]
Thailand	NA	30.0% (6/20)	6.75–23.57 mg/unit	Phattanawasin et al., 2012 [31]
Iran ^b^	NA	100% (9/9)	5400–781,200	Shekari et al., 2018 [32]
China	NA	22.5% (27/120)	260–113,220	Cheng et al., 2017 [33]

^a^ Sibutramine concentrations in the positive samples. ^b^ Samples were selectively sampled from unauthorized stores. NA: not available.

## 3. Plasticizer Contamination in Food Products

A major food scandal was reported in Taiwan in 2011, following the discovery of plasticizer-contaminated foods being sold in the market. Industrial plasticizers applied to increase the flexibility of a plastic material were deliberately substituted for food-grade emulsifiers in various food products and supplements [34,35,36]. The investigations traced back to two major providers in Taiwan. The companies had deliberately incorporated di-(2-ethyl-hexyl) phthalate (DEHP) and di-isononyl phthalate (DINP), and trace amounts of other phthalates, into palm oil and clouding agents used in food emulsification [36,37]. Plasticizer-tainted clouding agents were dispatched to 186 food ingredient providers and 229 end-product providers, which resulted in the contamination of a wide range of food products that were widely distributed in the Taiwanese market [38]. Furthermore, this scandal involved the export of 206 adulterated food items by 34 manufacturers to a total of 22 countries, including China, Hong Kong, Vietnam, the Philippines, Malaysia, Indonesia, Brunei, and Japan [36].

Phthalates are not approved for use in food formulations and are known to negatively affect the synthesis, secretion, binding action, and metabolism of sex and thyroid hormones, directly interfering with reproductive systems and neurodevelopment. These endocrine-disrupting chemicals display anti-androgenic and estrogenic effects, and were shown to cause male reproductive system abnormalities in rodents. Prolonged menstrual cycles, increased premature menopause, alterations with the reproductive tract, interference with kidney function, and the inducement of tumorigenesis have been observed in several animal models [35,38,39].

Studies on the Taiwanese population have revealed a correlation between DEHP exposure and lower testosterone levels, sperm DNA damage, poor quality semen, and shortened anogenital distance in men. In women, it was linked to early puberty, breast cancer, and endometriosis [40]. While it has not clearly affected neurodevelopment, one study has suggested the possibility of DEHP exposure being linked to attention deficit and hyperactivity disorder in children. Respiratory symptoms of allergic rhinitis and bronchial asthma were also diagnosed. These findings suggest that phthalate ingestion has adverse impacts on human health, and exposure from food is a major concern [40,41,42].

Many of the DEHP and DINP-tainted food items, including vitamins, anti-allergy supplements, and sports drinks, were frequently consumed by children. The highest concentration of DEHP (2108 parts per million) was found in a popular probiotic regularly fed to infants and children. An intake estimation for children weighing 20 kg taking one tablet of the probiotic product would exceed the tolerable daily intake (TDI) of DEHP of 0.05 mg/kg body weight/day [36,43]. According to an estimation of the DEHP intake of 60 kg adolescents, drinking one bottle of sports drink (350 mL) with 10 parts per million DEHP daily would result in a DEHP intake of 0.058 mg/kg body weight/day, exceeding the safety level [36].

Increased phthalate exposure is linked to a number of adverse health effects in the human population. A cohort study of Taiwanese children showed a correlation of increased phthalate exposure with decreased levels of testosterone and altered androgen-responsive brain development. Furthermore, decreased pre-school activities and masculinity scores were noted [44]. A few studies on the phthalate exposure from the 2011 Taiwan food scandal found that higher DEHP intake is associated with increased risks of microalbuminuria in children [35,45]. Altered concentrations of reproductive hormone and sex hormone-binding globulin (SHBG) levels were also observed in girls with higher DEHP exposure from the incident [46]. Other findings have also suggested an association with early puberty, endometriosis, adenomyosis, and leiomyoma in females. Adverse effects on males include decreased semen quality and sperm concentration, reproductive tract abnormalities, and testicular cancer [38,47]. High DEHP exposure in pregnant women was associated with poor birth outcomes. The presence of phthalates was linked to altered thyroid hormone and growth hormone levels, directly interfering with the thyroid secretion homeostasis required for normal fetal growth [35].

The immediate response of the Taiwan Food and Drug Administration (TFDA) to protect public health was to track down all tainted products and remove them from the shelves. Products underwent rapid screening for phthalate plasticizers in 49 private and government laboratories in Taiwan. Manufacturers of foodstuffs categorized as containing clouding agents were required to provide a safety certificate as proof of being plasticizer-free. Manufacturer and vendor inspections were carried out nationwide, in which 29,337 items from 406 stores were removed. The International Food Safety Authorities Network of the World Health Organization, the RASFF, and 22 countries involved in the exportation of tainted food items were notified of the incident by the TFDA, and were urged to take immediate action. Authoritative certificates became a mandatory requirement for the exportation of food products in the involved categories in June 2011.

The Taiwan Department of Health developed a system for the registration of food additives to allow for more comprehensive tracking and management, along with strict regulations for selling phthalate plasticizers. Suppliers now need to go through a detailed application process prior to obtaining approval to sell the items [37]. The chemicals were reclassified as toxic to the environment and human health; thus, manufacturers must report how these products will be distributed.

In attempts to educate and provide consultation to the Taiwanese population, information on the tainted products was publicized and updated daily on the TFDA website, along with health risk information. Hotlines and clinical consultations were established to address questions and concerns, and to provide health screening and track health effects [36,37]. Following this large-scale scandal, the DOH launched risk communication campaigns for the general public to reduce plasticizer exposure. The DOH posted on its media and distributed flyers containing information on how people can avoid exposure to these chemicals [36,37].

## 4. Gutter Oil

“Gutter oil” or “swill-cooked oil” are common terms used in China to explain illicit cooking oil that has been recycled from waste cooking oil collected from restaurant fryers, drains, and grease traps, as well as oil from slaughterhouse waste [48]. Gutter oil undergoes a series of simple processes that include collection, filtration, boiling, and refining. The clear oil produced from gutter oil is distributed to retailers, restaurants, and consumers [48].

Various chemical hazards, such as polycyclic aromatic hydrocarbons (PAHs), polychlorinated biphenyls (PCBs), and dioxins, could occur during the process of oil recycling [48,49]. Non-volatile substances, i.e., trans-4-hydroxy-2-nonenal (4-HNE), a major product of lipid peroxidation, was detected in heated cooking oil, especially vegetable oils with high levels of polyunsaturated fatty acids [50]. A previous study reported high contents of 16 PAHs in waste cooking oil collected from local markets in China [51]. A previous study reported that levels of PAHs in cooking oil from snack street vendors in China ranged between 2.8 and 532.0 parts per billion, in which the benzo[a]pyrene (BaP) contents in some samples exceeded the limit of 10 parts per billion of the Chinese Food Safety National Standard [52].

Evidence on adverse health effects due to the reuse of heated oil in humans is scarce; however, several animal studies have demonstrated detrimental effects of the intake of repeatedly heated oil [53]. In a study on the adverse effects of recycled cooking oil using a mouse model, recycled oil or fresh cooking oil (control group) were administered daily via oral gavage for 34 weeks at the dose of 0.1 mL/20 g body weight per day. The results revealed that damage to multiple organs (liver, kidneys, and intestines) occurred in mice fed repeatedly heated cooking oils (RHCO). The intake of RHCO may induce DNA damage in the cells, and consequent apoptosis [54]. Although the RHCO used in the animal study contained undetectable or low levels of some toxicants, i.e., aflatoxin, BaP, As, Pb, pesticides, trans-fatty acids, and polar compounds, the authors suggested that the toxic effects might be due to the combination of toxicants or unidentified toxic compounds present in RHCO [54]. A study conducted in mice using three doses (5, 10, and 20 μL/g body weight) of gutter oil or fresh cooking oil (control) were given to mice for 8 weeks. Significantly higher levels of alanine aminotransferase, aspartate aminotransferase, serum creatinine, and blood urea nitrogen were found in the gutter oil groups compared to the control. The acid and peroxide values of the gutter oil used in the animal study exceeded the national standard. The authors concluded that the intake of gutter oil for two months could damage liver and renal functions [55].

Enforcing a legal system for gutter oil is a major mitigation strategy for the gutter oil problem. To prevent the entering of illicit gutter oil into the food supply chain, to protect people’s health, three regulations have been implemented in China: “Strengthening the Prohibition of Gutter oil in the Catering Industry”, “Administrative Measures for Food Safety of the Catering Industry”, and “Administrative Measures for License of the Catering Industry” [48]. These regulations clearly state that people producing edible oil from gutter oil will be convicted for “adding toxic and hazardous non-food items in producing and selling food” under “Criminal Law”. However, law enforcement requires the systematic supervision and inspection of cooking oil that is acquired and used in restaurants and the catering sector.

Innovative, reliable, and highly sensitive methods are needed to respond to the gutter oil problem. The Chinese standard for edible vegetable oil, with only nine conventional quality indicators, allows gutter oil to enter the food supply chain. A previous study proposed a rapid technique to detect five long-chain aliphatic aldehydes. Higher levels of these long-chain aldehydes are present in gutter oil compared to fresh cooking oils [56].

An innovative analytical technique to distinguish gutter oil was developed based on the quantification of capsaicinoid compounds. Pepper and chili are spices commonly used in Chinese cuisine; therefore, heat-stable compounds of these spices can be used as indicators for reprocessed gutter oil [57,58]. Capsaicinoids, especially capsaicin, dihydrocapsaicin, and N-vanillylnonanamide, are the key compositions of pepper and chili, and are commonly detected in gutter oil [57]. These compounds are thermally stable, lipophilic, have a high boiling point, and cannot be removed via the processing of gutter oil.

Recent studies have shown that metabolomic and metabonomic approaches can be used to identify fraudulent food [59,60]. In addition to reliable, highly sensitive, and advanced instruments, powerful statistical software tools dealing with a number of experimental data are also needed [59,60]. In the near future, a metabonomic approach in combination with sophisticated statistical software could be a novel approach for the identification of complicated food adulterations, such as gutter oil.

The public concern regarding the gutter oil scandal has not been adequately addressed, and education of all stakeholders using different approaches is required. In response to the gutter oil issue in China, various communication strategies have been implemented, including communication regarding relevant laws for cooking oil, improving public health and environmental consciousness, raising the public’s awareness of the adverse effects of gutter oil, enforcing food safety training among employees in the food supply chain, and cultivating food safety and legal consciousness among food enterprises [48,61].

## 5. Adulterated Milk

Milk and dairy products are essential parts of the human diet, but are also susceptible to adulteration. Previous studies have reported that dairy products, particularly milk, are ranked in the top 10 food categories with the most reported incidences of fraud [62]. The most serious case of milk adulteration was the addition of melamine to milk in China in 2008, resulting in a number of infants suffering from renal failure, which has been reviewed and published on widely [63]. Therefore, this review article focuses on other cases of adulterated milk, especially in India, a major global dairy producer. Although some milk adulteration practices, such as adding water, whey, or vegetable oil to increase the volume of milk and/or boost the protein content, may not pose risks to consumers, adding toxic chemicals can cause serious health effects. Chemical adulterants of concern include urea, boric acid, salicylic acid, and formalin [64].

Urea is added to milk to the increase the protein (nitrogen) content. Urea is also added to milk to boost the content of solid-not-fat to meet the standard for milk quality. Because urea is colorless, odorless, and highly water soluble, this adulterant is commonly used in milk fraud [65]. A high intake of urea-containing milk may cause renal failure because it overburdens the kidney to filter out urea from the body. Other adverse effects include indigestion, diarrhea, intestinal tract and digestive system malfunctions, ulcers, and impaired vision [66,67]. Boric acid, salicylic acid, and formalin are harmful chemicals which may have fatal health consequences for consumers, especially vulnerable populations such as infants and young children [68]. These adulterants are not permitted to be used in foods; however, misuse of these chemicals has been reported, particularly in developing countries. The acute toxicities and symptoms reported in humans after high doses of boric acid intake include vomiting, headache, diarrhea, erythematous rash, tachypnoea, tachycardia, hypotension, renal failure, metabolic acidosis, and death [65]. The toxicity endpoints in animals are weight loss and reproductive toxicity. Studies on the toxicity of salicylic acid have demonstrated reproductive toxicity in various animal species [69]. The administration of higher doses of salicylic acid in rodents caused fetal malformations (skeletal malformations and growth retardation) and perinatal death. Health risks such as gastric irritation, bleeding, diarrhea, and death can occur after high dose ingestion [65]. The illegal use of formalin as a preservative has been reported in seafoods, vegetables, fruits, and milk in many developing countries in Asia [70]. Formalin is an aqueous solution containing approximately 37–50% dissolved formaldehyde (CH_2_O). Formaldehyde is classified as a class 1 human carcinogen by the International Cancer Research Agency. Acute toxicities of formalin include nausea, vomiting, diarrhea, bloody stool, breathlessness, vertigo, circulation failure, damage to the digestive system, and death [71].

Adulterated and synthetic milk scandals in India have been widely reported in the mass media, with supporting evidence from the government and scientific publications [72,73]. A national survey on milk adulteration conducted in India in 2011 reported that 68.4% of milk samples did not comply with the Food Safety and Standards Authority of India [74]. A previous study using 50 milk samples from Hyderabad, India, revealed that 60% and 32% of the samples were contaminated with urea and formalin, respectively [75]. An investigation revealed that urea was detected in 8% and 40% of milk samples from 365 households in Uttar Pradesh in India, gathered from rural and urban regions, respectively [66]. The most common symptoms reported among children consuming adulterated milk were headaches, diarrhea, and eyesight problems. Up to 52% of urban children suffered from diarrhea and eyesight problems, which could have been due to the concentrations of urea and detergents in the adulterated milk they had consumed [66].

A supportive approach for risk management, together with law enforcement, should be developed and implemented in collaboration with the private sector to minimize food adulterations. For example, a certification system such as good agricultural practice (GAP) for livestock farms, good manufacturing practice (GMP) for dairy plants, and good hygienic practice (GHP) for distributors could be implemented. The government should support the integration of small livestock owners and food traders into food safety and quality networks in order to facilitate the development of a traceability system [76]. Rapid and reliable methods for the determination of the presence of common milk adulterants, namely urea, boric acid, salicylic acid, and formalin, in milk are available [65]. However, the identification of some other toxic substances used for milk adulteration is very complicated. Untargeted approaches (screening for unknown adulterants) using chromatographic and spectroscopic techniques, combined with multivariate data analysis, have increased in the detection of food frauds [72].

A mechanism for attracting more public–private–people partnerships to enhance awareness, information access, sensitization, and capacity building for risk communication should be set up and implemented in relevant countries to overcome the milk adulteration problem [76,77]. Consumer organizations should greatly contribute to public communication via mass media about possible prevention strategies and health risks related to adulterated milk [74,76]. A rapid alert system for food-borne hazards at the district and state levels should be developed. Integrating small farmers and traders into food safety and quality networks by establishing a greater number of co-operatives may improve traceability systems [76]. Education and training courses for building awareness about food safety should be provided to all stakeholders in the food chain, including farmers, middlemen, transporters, food industries, distributors, retailers, and consumers. Risk communication and food education play a vital role in fostering safe agri-business development, both in the domestic and international trade markets [76,77].

## 6. Food Fraud Vulnerability Assessment

Conventional risk assessments of food hazards (biological, chemical, and physical agents with harmful potential), food allergens, and food quality concerns have been conducted independently. Existing food safety management systems have primarily been developed to address conventional food safety hazards, without a specific focus on food fraud prevention and control [78]. However, there has been a recent shift toward integrating various risk assessments, including food fraud vulnerability assessments (FFVA), into industry standards for food safety and quality [79]. Fraud vulnerability refers to vulnerabilities within the system that create opportunities for fraudsters to exploit. It is defined by three core elements: opportunities, motivations, and control measures [80]. The Global Food Safety Initiative (GFSI) has incorporated “VACCP” (Vulnerability Assessment and Critical Control Points) as one of the tools for mitigating food fraud [79]. The primary objective of VACCP is to identify and manage vulnerabilities within the food supply chain, particularly related to food fraud. This process systematically aims to prevent potential food adulteration, whether intentional or unintentional, by identifying weak points within the supply chain [79].

Several challenges in implementing FFVA have been identified by food operators, primarily due to the absence of a universally validated global framework for preventing food fraud [81]. Furthermore, there is a noticeable lack of comprehensive data concerning documented instances of fraud within the food industry, and the extent of the industry’s exposure to fraudulent activities. The limited emphasis placed on fraud as a policing priority has led to a significant backlog of uninvestigated cases [79]. Another contributing factor to food industry operators’ failure to adopt measures for mitigating food fraud is a shortage of both human resources and financial capacity [81]. Consequently, advocating for a culture within the food industry that rigorously examines the origins of its supply chain and upholds broader food integrity is essential.

## 7. Conclusions

Adulteration in the food industry remains a major worldwide concern. Food fraud has negative consequences for public health and the economy, destroying not only consumer confidence, but also the reputation of the country. Economic gains are the main drivers of food adulterations. Adulteration methods are complex and difficult to assess based on the product information declared by manufacturers. Vigorous control and the inspection of raw materials, as well as an effective system to monitor food handling, processing, and distribution systems, are required to minimize food fraud. The enforcement of food safety regulations and good manufacturing practices by governments, targeting all stakeholders involved along the food supply chain, will reduce the occurrence of fraudulent practices. Establishing a global real time alert system is critical for safeguarding the food industry from fraud and adulteration, and reducing public health and safety risks. To foster long-term prevention, it would be beneficial for underlying economic issues to be addressed and resolved at the national and/or international level.

## Figures and Tables

**Table 1:** Number of fraud and adulteration cases of products imported from Asia between 2011 and 2020, recorded in the RASFF database.

Country/Region	Cases	Country/Region	Cases
China	200	Hong Kong	5
India	172	Myanmar	5
Turkey	117	South Korea	4
Iran	37	United Arab Emirates	3
Japan	28	Taiwan	3
Thailand	22	Pakistan	2
Indonesia	18	Azerbaijan	2
Vietnam	18	Sri Lanka	2
Bangladesh	16	Kazakhstan	1
Philippines	9	Oman	1
**Asian-originated products**			**663**
non-Asian-originated products			503

**Table 2:** Number of reported food fraud and adulteration cases by product type.

Product Type	Number of Cases
Nuts, nut products, and seeds	189
Fruits and vegetables	96
Herbs and spices	89
Cereals and bakery products	41
Confectionery	29
Fish and fish products	29
Dietetic foods, food supplements, and fortified foods	27
Other food product/mixed	26
Prepared dishes and snacks	20
Cocoa and cocoa preparations, coffee, and tea	16
Poultry meat and poultry meat products	16
Soups, broths, sauces, and condiments	16
Cephalopods and products thereof	15
Milk and milk products	10
Eggs and egg products	7
Honey and royal jelly	7
Bivalve mollusks and products thereof	6
Meat and meat products (other than poultry)	5
Non-alcoholic beverages	5
Crustaceans and products thereof	4
Food additives and flavorings	3
Natural mineral water	3
Alcoholic beverages	2
Fats and oils	2
**Total**	**663**

## Data Availability

The datasets generated for this study are available on request to the corresponding author.
